# Progress in Stem Cell Therapy for Spinal Cord Injury

**DOI:** 10.1155/2020/2853650

**Published:** 2020-11-05

**Authors:** Liansheng Gao, Yucong Peng, Weilin Xu, Pingyou He, Tao Li, Xiaoyang Lu, Gao Chen

**Affiliations:** Department of Neurosurgery, Second Affiliated Hospital, School of Medicine, Zhejiang University, Hangzhou, Zhejiang, China

## Abstract

**Background:**

Spinal cord injury (SCI) is one of the serious neurological diseases that occur in young people with high morbidity and disability. However, there is still a lack of effective treatments for it. Stem cell (SC) treatment of SCI has gradually become a new research hotspot over the past decades. This article is aimed at reviewing the research progress of SC therapy for SCI.

**Methods:**

Review the literature and summarize the effects, strategies, related mechanisms, safety, and clinical application of different SC types and new approaches in combination with SC in SCI treatment.

**Results:**

A large number of studies have focused on SC therapy for SCI, most of which showed good effects. The common SC types for SCI treatment include mesenchymal stem cells (MSCs), hematopoietic stem cells (HSCs), neural stem cells (NSCs), induced pluripotent stem cells (iPSCs), and embryonic stem cells (ESCs). The modes of treatment include in vivo and in vitro induction. The pathways of transplantation consist of intravenous, transarterial, nasal, intraperitoneal, intrathecal, and intramedullary injections. Most of the SC treatments for SCI use a number of cells ranging from tens of thousands to millions. Early or late SC administration, application of immunosuppressant or not are still controversies. Potential mechanisms of SC therapy include tissue repair and replacement, neurotrophy, and regeneration and promotion of angiogenesis, antiapoptosis, and anti-inflammatory. Common safety issues include thrombosis and embolism, tumorigenicity and instability, infection, high fever, and even death. Recently, some new approaches, such as the pharmacological activation of endogenous SCs, biomaterials, 3D print, and optogenetics, have been also developed, which greatly improved the application of SC therapy for SCI.

**Conclusion:**

Most studies support the effects of SC therapy on SCI, while a few studies do not. The cell types, mechanisms, and strategies of SC therapy for SCI are very different among studies. In addition, the safety cannot be ignored, and more clinical trials are required. The application of new technology will promote SC therapy of SCI.

## 1. Introduction

Spinal cord injury (SCI) is one of the most serious neurological diseases in the world. Due to the high disability rate, SCI brings a high economic burden to the society [1]. It is reported that the incidence of SCI in the young population is relatively high, but in recent years, its incidence in the elderly population has also shown a gradual upward trend [2]. Despite the studies focused on SCI have been carried out for decades, the surviving patients of this disease will inevitably leave long-term and severe neurological damage [3]. According to previous researches, there are many risk factors for traumatic SCI including violence, extreme sports, and drunk driving [4]. Studies have reported that more than 23% of SCI patients have had secondary injuries within 10 years. Alcoholism, the use of psychotropic drugs, and some personality traits are all related to the occurrence of secondary injury [5].

The pathological mechanism of SCI can be divided into two processes: primary injury and secondary injury. When the spinal cord is subjected to contusion, tearing or compression due to external forces, or infarction due to vascular injury, the spinal cord begins to have nerve damage, which is often referred to as primary injury [6]. After the primary injury occurs, a large number of nerve cell death occurs, and the blood-spinal cord barrier is destroyed. Subsequently, a series of damage reactions such as vasospasm hemorrhage, reactive oxygen species (ROS) formation, lipid peroxidation, inflammation, and apoptosis occur, leading to secondary cascade reaction which further aggravates the damage [7]. At present, the main methods for the treatment of SCI include surgical treatment, drug treatment, hyperbaric oxygen therapy, and physical therapy. However, the therapeutic effects and the outcome of patients with SCI remain unsatisfactory. [8]. This requires us to study new and effective methods for the treatment of SCI. In recent years, stem cell (SC) treatment of SCI has gradually become a new research hotspot.

SCs refer to cells that have the ability to proliferate and self-renew under certain conditions and differentiate into many other functional cells [9]. There are two ways to classify stem cells. According to different developmental stages, SCs can be divided into embryonic stem cells (ESCs) and adult stem cells (ASCs), in which ESCs are separated from the blastocyst cell cluster; ASCs exist in various adult tissues and can be divided into mesenchymal stem cells (MSCs), hematopoietic stem cells (HSCs), neural stem cells (NSCs), induced pluripotent stem cells (iPSCs), and the like. According to their differentiation potential, SCs can be divided into totipotent stem cells (TSCs), pluripotent stem cells (PSCs), and unipotent stem cells (USCs). People have tried to use SCs to treat human diseases for decades. The most typical example is the use of SC transplantation to treat a variety of malignant or benign blood diseases. This technology is now mature in the field of hematology and has been widely used, with great clinical value [10]. In recent years, SC therapy for varied neurological diseases, such as intracerebral hemorrhage (ICH), ischemic stroke, traumatic brain injury (TBI), and subarachnoid hemorrhage (SAH), is also developed [11–14]. At present, more and more animal experiments and clinical trials show that the use of SCs to treat SCI can play a beneficial therapeutic effect. SC therapy has great potential in saving damaged tissues and promoting nerve function recovery [14]. MSCs, HSCs, NSCs, iPSCs, and ESCs are the most common types of SCs used for the treatment of SCI.

Recent studies have shown that the possible therapeutic mechanisms of SC therapy for SCI involve multiple aspects [15]. SC transplantation can repair or replace damaged nerve cells and tissues, including neurons and glial cells, which helps to ensure the integrity of the nerve conduction pathway and thereby reconstruct nerve function [7]. At the same time, SCs interact with surrounding tissues to produce a variety of neurotrophic factors, altering the microenvironment of the injured site, and accelerating the growth of axons, while interneurons differentiated from transplanted SCs can cause axon sprouting, and the proximal and distal ends of the spinal cord are connected to the injury site to induce the formation of new synapses [16]. After SCI, SC transplantation can downregulate genes involved in inflammation and apoptosis as well as upregulate genes with neuroprotective effects, thereby protecting spinal neurons from secondary damage [17]. Some transplanted SCs can differentiate into glial cells and promote myelination and functional recovery in patients with SCI [18].

Notably, the disadvantage of SC transplantation such as lack of donors, rejection reaction, and ethical factors limits the application of SC in SCI treatment. Therefore, some scholars have focused on mobilizing SCs in SCI patients themselves, intending to treat SCI while avoiding the common problems of SC transplantation. Moreover, new approaches including scaffolds, 3D print, and optogenetics have been introduced to enhance the therapeutic effect of SC on SCI.

The current research on the use of SC to treat SCI remains to be deepened. This review is intended to provide a detailed summary of the neuroprotective effects of SC and the underlying mechanisms and related issues of SC therapy for SCI. SC is expected to be applied to the treatment of SCI in the near future.

## 2. Common SC Types for SCI Treatment

### 2.1. ESCs

Whether in vitro or in vivo, ESCs can be induced to differentiate into almost all cell types, including neurons and glial cells, making them one of the most promising SCs for the treatment of central nervous system (CNS) diseases [19, 20]. ESCs can express neuron-specific antigens by all-trans retinoic acid induction, some of which may have glial-specific antigens, while some neuron-like cells may even have acetylcholinesterase or glutamate decarboxylase activity. [21]. In recent years, there have been many reports on the use of ESCs to differentiate into neurons and glial cells for the treatment of SCI [22–24]. Manley et al. injected human embryonic cell-derived oligodendrocyte progenitor cells into the injury site of SCI nude mice and found that progenitor cells can migrate to the spinal cord and brain stem, thus decreasing the parenchymal cavity of the injury site, promoting the survival of axons, and improving the motor function of nude mice, without causing adverse reactions, such as pain, toxicity, and tumor [25]. Hwang et al. used ESCs to induce the differentiation of spinal GABAergic neurons and injected them intrathecally into SCI rats. The results suggest that ESC-derived spinal GABAergic neurons significantly reduce chronic neuropathic pain after SCI [26].

### 2.2. MSCs

The most commonly used MSCs in clinical practice are bone marrow mesenchymal stem cells (BM-MSCs), human umbilical cord mesenchymal stem cells (HUC-MSCs), and adipose-derived mesenchymal stem cells (AD-MSCs).

BM-MSCs have been reported to have a therapeutic effect on a variety of diseases, including stroke. The researchers have proved that transplanted BM-MSCs can pass the blood-brain barrier (BBB) without destroying their structure [27]. After transplantation, BM-MSCs can migrate to the injured area and differentiate into neurons or neuron-like cells, thereby exerting neuroprotective effects by secreting various neurotrophic factors [28–31]. Many studies have shown that BM-MSC transplantation can alleviate neurological deficits in SCI rats and promote the recovery of their neurological functions [32–42]. Gu et al. reported that BM-MSCs can improve motor function after SCI in rats and reduce the expression of CHOP, thereby reducing apoptosis [43]. Zhou et al. reported that BM-MSCs can promote the production of unmyelinated and myelinated nerve fibers in the spinal cord of SCI rats, resulting in a significant improvement on the motor function of mice [44]. Han et al. reported that BM-MSCs can inhibit TLR4-mediated signaling and reduce interleukin-1*β* (IL-1*β*) and tumor necrosis factor-*α* (TNF-*α*) expression to alleviate the inflammatory response after SCI and improve neurological function in rats [45].

HUC-MSCs have also been used to treat SCI in animals and patients [46–53]. Yousefifard et al. showed that HUC-MSC transplantation can alleviate the symptoms of neuropathic pain and can promote the recovery of motor function after SCI; they also proposed that rat survival and electrophysiological monitoring results were significantly better after HUC-MSC transplantation than BM-MSC transplantation [54]. Zhilai et al. pointed out that HUC-MSC transplantation can reduce the number of caspase-3 positive cells and ED-1 positive macrophages at the injury site and promote the survival of axons in a rat SCI model, [49]. Clinically, Zhao et al. implanted a nerve regeneration scaffold containing HUC-MSCs into the injury site of chronic SCI patients, founding that this measure can promote the regeneration of damaged neurons and improve the sensory and motor function after complete SCI, and the security is better [55].

There are many studies on the therapeutic effects of AD-MSCs for various diseases including ICH [56–58]. Kim et al. showed that early injection of AD-MSCs in adult dogs after acute SCI can prevent further damage by enhancing antioxidant and anti-inflammatory mechanisms, and no adverse reactions were found [59]. Hur et al. conducted a clinical trial involving 14 patients with SCI. Isolated autologous AD-MSCs from the liposuction of subcutaneous adipose tissue were injected intrathecally into the patient via lumbar puncture. Magnetic resonance, hematology, electrophysiological examination, and motor sensation scores were tested before and 8 months after transplantation. The results showed that autologous AD-MSCs had no significant adverse reactions in the treatment of SCI, and some patients had a slight improvement in neurological function [60].

### 2.3. HSCs

In recent years, more and more studies have begun to focus on the application of HSCs in the treatment of SCI [61–63]. Xiong et al. injected HSCs into a rat model of SCI and found that HSCs can promote the formation of 5-HT-positive fibers and oligodendrocytes in the spinal cord, inhibit astrocyte hyperplasia, and upregulate neurotrophins-3 (NT-3) mediated MEK-1 expression, thereby promoting neurological recovery in rats [64]. Frolov and Bryukhovetskiy used HSC therapy in 20 patients with C4-C8 intermittent chronic SCI and used somatosensory evoked potentials and motor evoked potentials during the treatment to find that HSCs can spread from the waist to the neck and play a nerve repair effect [65]. Al-Zoubi et al. transplanted CD34 and CD133-positive SCs directly into the injured area of 19 patients with thoracic SCI. After 5 years of follow-up, the safety and efficacy of SC therapy were evaluated. Seven patients were found to have segmental sensation improvements, 2 patients showed improvement in motor function, and no adverse reactions occurred in all patients [66].

### 2.4. NSCs

Numerous studies have shown that NSC transplantation can promote the recovery of neurological function after SCI [7, 67–70]. Endogenous NSCs are normally silenced; however, they can be activated under a variety of pathological conditions and migrate to the site of injury to promote nerve repair [71]. Liu et al. used Dil-labeled endogenous NSCs to track the differentiation of cells after mild SCI. It was found that rat SCI can induce proliferation and differentiation of endogenous NSCs [72]. Cheng et al. found that NSC transplantation can modulate SCI-induced inflammatory responses and improve neurological function after SCI by reducing M1 macrophage activation and neutrophil infiltration [73]. You et al. found in vitro and in vivo studies that neuronal cell-specific gene expression systems can induce overexpression of granulocyte-macrophage colony-stimulating factor (GMCSF) in NSCs and exert neuroprotective effects. Thus, a neural cell-specific gene expression system and NSCs can be used in combination to treat SCI [74].

### 2.5. iPSCs

The use of iPSCs to treat SCI is still in the experimental stage [75–77]. Lu et al. transplanted iPSCs from 86-year-old healthy males into immunodeficient rats after SCI and found that iPSCs survived and differentiated into neurons and glial cells and extended tens of thousands of axons from the injury site, which cover almost the entire rat CNS [78]. Oh et al. obtained iPSCs from intervertebral disc cells after SCI and then transplanted them into SCI mice. It was found that the hind limb motor dysfunction of the experimental mice was significantly improved. This study gave us a new idea of the cell source of iPSCs [79]. Qin et al. conducted a meta-analysis of the effects of iPSC transplantation on the motor function of SCI rats. It was concluded that iPSC transplantation can significantly improve the recovery of motor function in SCI rats, demonstrating that iPSCs have certain application prospects for SCI treatment [80]. However, iPSC transplantation also has some drawbacks, such as low survival rate and possible tumor formation at the transplant site. Fuhrmann et al. found that in the SCI model, injection of hydrogel promoted the early survival of iPSC-derived oligodendrocytes and reduced the formation of teratoma [81].

### 2.6. Other SCs

Recent studies have shown that dental pulp stem cells (DPSCs) have the potential to differentiate into neural-like cells and myocyte-like cells. Notably, a growing number of studies indicated the role of DPSCs in SCI treatment. Upon being transplanted into the lesion site, DPSCs can differentiate into Schwann-like glial cells, secreting neurotrophic factors (NTF) and promoting survival and neurite outgrowth in a rat SCI model [82]. Martens et al. reported that DPSCs could promote axon regeneration and survival of endogenous neurons and glia within and around the lesion site through a paracrine-mediated mechanism [83]. And the administration of DPSCs with biomaterials such as engineered 3D scaffolds and DPSC/chitosan scaffold has been reported to enhance the effect of DPSCs in treating SCI via providing mechanical support to promote cell adhesion, migration, and in vivo differentiation [84, 85].

The transplantation of olfactory ensheathing cells (OECs) on CNS injury treatment including SCI has been gradually revealed over the past decades [86, 87]. OEC transplantation has emerged as a promising repair strategy due to the feature of modulating the host environment to promote remyelination [88]. Zhang et al. reported that intravenous transplantation of OECs conferred a robust neuroprotection against SCI via suppressing the neuroinflammation, evidenced by the decreased number of activated microglia and upregulated anti-inflammatory cytokines such as IL-4 and IL-10 in a rat model [89]. In addition, Wright et al. reported that activating OECs with neurotrophins could enhance the therapeutic potential of OECs in spinal cord repair and improve neurological recovery [90]. Besides, in rodents, the beneficial effect of OECs has also been confirmed in human patients [91]. Moreover, Czyz et al. reported a minimally invasive procedure to harvest the olfactory bulb OECs in human subjects, which significantly increased the safety of the application of OECs in SCI treatment [92]. More importantly, Liadi et al. have demonstrated that storing olfactory bulb tissue before culture could be achieved without compromising the viability of cells, which makes it possible to obtain a large number of cells for the clinic use of autologous, particularly allogeneic and OEC transplantation [93].

**Figure 1:**
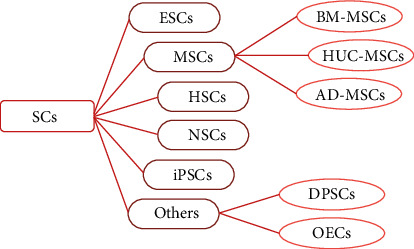
The common stem cell (SC) types for the treatment of spinal cord injury (SCI), including embryonic stem cells (ESCs), mesenchymal stem cells (MSCs), hematopoietic stem cells (HSCs), neural stem cells (NSCs), induced pluripotent stem cells (iPSCs), dental pulp stem cells (DPSCs), and olfactory ensheathing cells (OECs). MSCs consist of bone marrow mesenchymal stem cells (BM-MSCs), human umbilical cord-mesenchymal stem cells (HUC-MSCs), and adipose-derived mesenchymal stem cells (AD-MSCs).

The common SC types for the treatment of SCI are shown in Figure 1.

## 3. Strategies of SC Therapy for SCI

### 3.1. Modes of Treatment

There are two main modes of SC transplantation: in vivo and in vitro induction. The former is to transplant the appropriate SCs directly into the body, and the in vivo environment and specific signaling molecules will guide these SCs into the desired mature cells to perform the necessary functions; the latter is to isolate, culture, purify, and amplify a certain SC, and induce it to differentiate into cells having a desired function in vitro, and transplant these mature cells into a human body for treatment. The proper combination of the two techniques may have the best effect on the patient.

### 3.2. Pathways of Transplantation

There are many pathways to transplant SCs in various SCI models, including intravenous, transarterial, nasal, intraperitoneal, intrathecal, and intramedullary injections [94]. It was concluded that various routes of SC administration was feasible for the treatment of SCI [95].

Intravenous administration is invasive and does not damage the spinal cord tissue, and the number of cells that can be administered at one time is also large. After being administered to SCI rats by intravenous annotation, NSCs can migrate to the site of SCI and then differentiate into neurons and glial cells, replacing damaged cells to treat SCI [96]. Ohta et al. injected AD-MSCs into the veins of SCI rats and observed that AD-MSCs gradually aggregated into the site of SCI, and the motor function of the rats also improved [97]. A small number of studies have used intra-arterial administration to study the therapeutic effects of SCs on SCI and found that SCs can also migrate to the injury site. However, there are also some drawbacks through intravascular administration, such as easy to cause blood vessel embolism.

Intramedullary injections are more effective at injecting SCs into the injured site than intravenous injection, whereas intrathecal injection is less invasive than intramedullary injection and can reduce the host's immune response [98, 99]. Levi et al. evaluated the safety of intramedullary injections for the treatment of chronic cervical and thoracic SCI. No adverse events associated with cell transplantation were found in 29 patients with cervical or thoracic SCI [100]. Amemori et al. compared the effects of intramedullary and intrathecal implantation of iPSC-derived neural precursors on SCI in rats and found that the cells survived for 2 months by intramedullary injection, but the cells injected by intrathecal injection were not detected at the site of administration or in spinal cord tissue; studies have found that intrathecal transplanted cells may have a mild therapeutic effect on SCI through a paracrine mechanism, while longer survival time of intramedullary cells may promote spinal cord tissue long-term regeneration [101].

Intranasal administration of bone marrow stromal cells can also cause them to migrate to the injured spinal cord and contribute to the reduction of the damage cavity and the recovery of hindlimb motor function; however, the therapeutic effect is not as significant as that of intrathecal administration [102]. Ramalho et al. compared the intraperitoneal and intravenous administration of BM-MSCs and found that the two approaches had similar therapeutic effects on SCI [103]. In summary, all of these pathways have proven to be relatively safe and with no major complications. However, the optimal route of administration has not yet been determined. Consideration should be given to the factors such as the type and number of SCs, patient characteristics, and more effective and safe route of administration should be researched.

### 3.3. Number of SCs

The number of SCs is an important issue affecting the therapeutic effect. An insufficient number of transplanted cells will make it difficult to exert therapeutic effects. Most studies of the SC treatments for SCI used a number of cells ranging from tens of thousands to millions and had significant therapeutic effects. Ramalho et al. used an 8 × 10^5^ number of MSCs to treat SCI mice by intravenous or intraperitoneal administration and found that the nerve fibers damaged in the spinal cord of the mice after administration were reduced, and the motor function of the mice was improved [103]. Hosseini et al. cultured NSCs through a medium containing alginic acid scaffold and injected them in a dose of 1 × 10^5^ for the treatment of SCI rats. It was found that inflammation and apoptosis were effectively inhibited after administration. The neurological function scores of the rats were also improved [104]. Compared with low doses, high-dose SCs can promote the differentiation of transplanted cells into neurons and the migration of transplanted cells to the distal end of the lesion by adjusting the expression of neurotrophic factors such as microtubule-associated protein 2 (MAP2) and artemin (ARTN) and achieve better nutrition and support to damaged tissue [105–107].

However, the number of cells used in SC therapy is not the more the better. Iwai et al. reported that after the number of transplanted SCs in mice after SCI exceeded a certain threshold, the number of SCs surviving after transplantation was basically the same, and there was no correlation with the number of transplanted cells [108]. Piltti et al. indicated that the number of SCs had no effect on terminal sensory recovery or motor score in SCI mice [109]. Studies based on the long-term efficacy of SC therapy for SCI showed that the effectiveness of autologous HSC transplantation is not directly dependent on the number of transplanted cells [110]. When the number of cells is very high, it will even have a negative impact on the proliferation of human cells [109], indicating that there are still some restrictions on the adaptation of SCI sites to a large number of transplanted cells.

In addition, depending on the number of cells, the transplanted cells may have different interactions with the microenvironment of the transplant target site to affect the differentiation of SCs. When pluripotent human CNS-derived NSCs were transplanted at a cell number of 10,000 (low) to 500,000 (high), they mainly differentiated into oligodendrocytes. However, as the number of transplanted cells increased, the proportion of oligodendrocytes differentiation decreased, both of which have been shown to be associated with decreased motor coordination function [109]. One possible explanation for these results is that the high number of transplanted cells in the high-dose group produces or integrates a large number of mature neurons, which can negatively regulate the spinal cord in the absence of external intervention, thereby affecting the expected therapeutic effect of SC treatment [111–113]. Therefore, the number of effective SCs for the treatment of SCI is still not very clear, which needs further study.

### 3.4. Time Window

Different studies have different perspectives on the time window of SC treatment of SCI, and some of them support the early use of SC transplantation for the treatment of SCI. Since SCI usually causes secondary damage such as inflammation and apoptosis within a week [114], early SC transplantation can effectively reduce the occurrence of secondary injury and promote the recovery of nerve function. The researchers found that early transplantation of BM-MSCs may reduce the acute inflammatory response after SCI, which may be related to changes in the inflammatory environment after transplantation [115]. At the same time, early transplantation of BM-MSCs can also regulate the activity of glial cells and blood-derived macrophages after SCI, reduce inflammation, relieve neuralgia after injury, and promote motor function recovery [116]. All et al. reported that the transplantation of iPSC-derived oligodendrocyte progenitor cells into SCI rats at an early stage reduced the glial scar and cavity volume at the injury site and observed that the transplanted cells could be observed to survive for 3 months [117]. In addition, some studies have shown that delayed intervention of SCs has a good effect on the treatment of SCI. Some scholars believe that a large number of neurotoxins will be produced in the early stage after SCI, which is not conducive to the survival of transplanted SCs, and SC transplantation from 1 week to 2 weeks after SCI can contribute to the recovery of nerve function [118]. Moon et al. reported that the administration of AD-MSCs after 3 weeks of spinal cord ischemic injury can exert neuroprotective effects by regulating microglia and brain-derived neurotrophic factor (BDNF) levels in the spinal cord [119]. In summary, the treatment time window of SCs varies with SC type, mode of administration, and animal model, and further study of a reasonable SC treatment time window is needed.

### 3.5. Application of Immunosuppressive Therapy

The immune barrier is a major obstacle to the clinical transformation of allogeneic SC transplantation therapy. The immunosuppressive regimen is continuously optimized and thus greatly increases the likelihood of successful transplantation. However, nonspecific immunosuppression may also cause various adverse consequences, including infection and malignancy, hypertension, diabetes, nephrotoxicity, and high blood lipid levels [120].

Studies have shown that allogeneic MSCs transplanted into the intact spinal cord of rats can survive in a short period of time, and immunosuppressive therapy prolongs their survival time [121], indicating the presence of immunogenicity of transplanted MSCs in vivo. After a few days of allogeneic MSC transplantation after SCI, the expression of immune-related genes was also detected, and the survival time of MSC grafts was prolonged by immunosuppression, indicating that the grafts were indeed rejected by the immune system [122]. In addition, human CNS-derived NSCs transplanted into immunodeficiency NOD-scid mice showed good survival and differentiation [123] in subacute [124] and chronic phase [125] after SCI, demonstrating that xenografts have a good therapeutic effect in the absence of xenograft rejection. Therefore, immunosuppressive therapy seems to be necessary for SC transplantation. Most in vivo and in vitro studies have shown inhibition of transplant rejection by immunosuppressive drugs and their promotion of cell survival [126]. These drugs reduce the inflammatory response activated by the traumatic SCI and promote the regeneration of the tissue and the rate of axonal branching [127, 128]. However, studies have shown that the beneficial effects of immunosuppressive agents on promoting graft cell survival and neurological recovery in SCI may be offset by other factors, such as the negative effects of immunosuppressive drugs on wound and spinal cord healing [129]. In addition, immunosuppressive drugs have been reported to affect the cellular behavior of SCs [130, 131]. Therefore, the current use of immunosuppressive agents in SC therapy for SCI and its specific details, such as the potential interaction of the inhibitor itself with the transplanted cells, still require further research to clarify.

## 4. Mechanisms of SC Treatment for SCI

### 4.1. Tissue Repair and Replacement

The translocated SCs could differentiate into neurons and glia cells under the stimulation of the internal environment and various nerve growth factors, which initiate the process of SCI repair and replacement [132]. After delayed transplantation of NSCs into the injured spinal cord of monkeys, it was observed that the transplanted NSCs survived and differentiated into neurons, astrocytes, and oligodendrocytes, and the injury lesion was reduced when compared with the control group. At the same time, the grip strength and autonomous exercise ability of the transplanted animals were significantly higher than those of the control group [133]. Zhao et al. induced and cultured primitive NSCs from human embryos. After they injected these primitive NSCs into the developing chicken CNS, the SCs integrated into the dorsal side of the neural tube and formed cell clusters, which differentiated into neurons. Upon migration into the injured spinal cord, these primitive NSCs (derived from both NSCs and ESCs) could differentiate into mature neurons and glia, forming a functional neural circuit around the spinal lesion and promote the restoration of axons [134]. In conclusion, the efficacy of SC therapy for SCI might be individualized due to the differences in the type of cells transplanted. In addition, more effort is required to better explore the mechanism of SCs in the repairing of the spinal cord in further.

### 4.2. Neurotrophic and Regenerative Effects

Nerve regeneration and neurotrophicity have been identified as important factors in the process of SCI repair and are also an important research direction of SCI treatment [135, 136]. Zhao et al. reported that mouse AD-MSCs could differentiate into neurogenic cells in vitro, and neurogenic cells were succeeded to survival and proliferation around the SCI lesion, evidenced by the increased level of neurogenic cell-specific markers including Nestin, GFAP, and MAP2 [137]. At the same time, there is increasing evidence that transplanted NSCs can release neurotrophic factors to achieve SCI treatment. Studies have shown that epidermal neural crest SCs can be transplanted into the in vitro SCI model, and valproic acid is given to improve the harsh injury environment of the transplant. Evaluation of the treated sections after 7 d postinjury shows that the expression of GFAP, BDNF, NT-3, and B-cell lymphoma-2 (Bcl-2) was significantly increased in the SC treated sections, indicating that NSCs can improve SCI by direct release of neurotrophic factors [138]. In addition, there is a large body of evidence that glial cell-derived neurotrophic factor (GDNF) can play a role in SCI repair [139]. In summary, after SCI, transplanted NSCs can play a key role in nerve regeneration and nutrition.

### 4.3. Promotion of Angiogenesis

The recovery of neurological function depends not only on the regeneration of nerve cells but also on the support of the surrounding microenvironment including blood vessels and extracellular matrix, which refers to the formation of new blood vessels that contribute to tissue repair. Vascular regeneration often occurs in neurological damaging diseases including SCI and is a valuable therapeutic research direction [140]. The researchers studied the effects of NSC transplantation on angiogenesis in SCI rats and performed BBB scores on rats at different time points after transplantation, and vascular endothelial growth factor (VEGF) was analysed by immunofluorescence and immunoblotting. The results showed that BBB score and VEGF protein expression in the transplanted group were significantly higher than those in the control group 14 days after transplantation. The results suggest that NSC transplantation can promote angiogenesis by inducing VEGF expression and improve limb motor function [141]. In addition, some researchers examined the effects of VEGF, angiopoietin-1, and basic fibroblast growth factor (bFGF) on angiogenesis, nerve regeneration, and neurological function in SCI rats and found sustained released angiogenic factors entered into the SCI site and significantly stimulated angiogenesis and nerve regeneration and accelerated neurological recovery [142]. The extracellular matrix is a supporting component of nerve tissue, and the MSC-derived fibronectin and cell adhesion molecules (integrin, cadherin, and selectin) in the extracellular matrix can promote nerve repair and axonal regeneration [143]. In summary, various nutrient factors and molecular components participate in the reconstruction of neurovascular units, which together promote the improvement of neurological function.

### 4.4. Antiapoptotic Effect

Apoptosis involves almost all neurological diseases including SCI, and it is also closely related to the recovery of neurological function. Gu et al. found that after transplanting MSCs into SCI rats, the number of terminal deoxynucleotidyl transferase-mediated dUTP nick-end labeling- (TUNEL-) positive cells was significantly lower than that of SCI alone, and the number of neurons in SCI rats was also significantly increased, and neurological function was significantly improved [43]. The researchers found that the proapoptotic proteins such as p53, caspase-9, caspase-3, and Bax were significantly downregulated compared with the simple SCI group by SC treatment, while antiapoptotic proteins such as Bcl-2 were significantly upregulated [144–148]. Nicola et al. detected neurons, astrocytes, macrophages/microglia, and T cells at different time points and tested the proapoptotic and antiapoptotic factors. The results indicated that the proapoptotic factor TNF-*α* was significantly downregulated in the SC transplantation group, while the antiapoptotic factor B-cell lymphoma-extra large (Bcl-xL) was upregulated when compared with the control group. The results suggest that SC transplantation can interfere with the balance between proapoptotic factors and antiapoptotic factors 1 h after SCI and reduce early neuronal apoptosis, thus contributing to the survival of tissues and motor neurons and the recovery of neurological function [149]. In conclusion, the antiapoptotic mechanism of SCI treated by SC transplantation has great research value.

### 4.5. Anti-Inflammatory Effect

Another important mechanism of SC transplantation for SCI treatment is anti-inflammatory effects [150–152]. Cheng et al. transplanted NSCs into SCI rats, neutrophils and macrophages were stained, and the mRNA levels of TNF-*α*, IL-1*β*, IL-6, and IL-12 were detected to analyze the anti-inflammatory effect. The results showed that NSC transplantation significantly reduced the number of neutrophils and iNOS+/mac-2+ cells in the injured area, and at the same time, the mRNA levels of TNF-*α*, IL-1*β*, IL-6, and IL-12 were significantly lower than those of the control group. These results indicate that NSC transplantation can modulate SCI-induced inflammatory responses and improve neurological function after SCI by reducing M1 macrophage activation and neutrophil infiltration [73]. In addition, the research team further found that NSC conditioned medium can improve the neurological function of SCI by inhibiting the inflammatory response, thus achieving the corresponding therapeutic effect [69]. In addition, Wang et al. found that decellularized spinal cord scaffolds implanted with BM-MSCs can repair spinal cord hemisection defects by regulating the recruitment of inflammatory cells and inhibiting apoptosis and secondary inflammatory responses, thereby promoting functional recovery [153]. In summary, after SC transplantation, it can exert anti-inflammatory effects and cooperate with other related mechanisms to promote tissue function repair after SCI.

**Figure 2:**
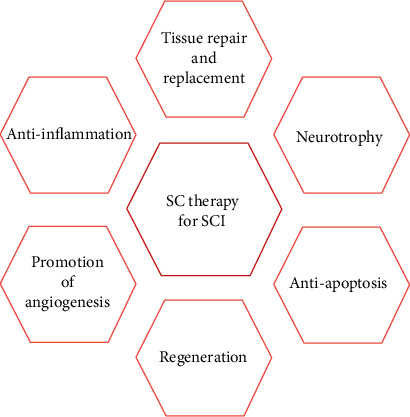
The mechanisms of stem cell (SC) therapy for spinal cord injury (SCI). The potential mechanisms of stem cell therapy for SCI include tissue repair and replacement, neurotrophy, regeneration, promotion of angiogenesis, antiapoptosis, and anti-inflammatory.

The mechanisms of SC treatment of SCI are shown in Figure 2.

## 5. Safety of SC Therapy for SCI

The safety and reliability of SC therapy for SCI cannot be ignored. Some studies have reported that excessive SCs or excessive infusion rates may cause thrombosis or embolism, leading to vascular occlusion [154, 155]. Other studies suggest that transplanted SCs can cause a certain degree of immune rejection, thus it is recommended to perform immunosuppressive therapy at the same time [118, 126, 156]. The most serious side effects of SC transplantation are tumorigenicity and instability [157–159]. Iida et al. found that the DNA methylation pattern of NSCs and progenitor cells derived from human-induced multifunctional SCs is not stable, and this instability gradually appears with passage [160]. Miura et al. found that mouse BM-MSCs can spontaneously transform into malignant cells and form fibrosarcoma in vivo, which may be related to chromosomal abnormalities, telomerase activity increase, and increased expression of c-Myc116 [161]. In addition, SC transplantation can cause other side effects such as infection, high fever, and even death [162–164]. Therefore, before using SCs to treat SCI, the safety issues associated with SC therapy should be carefully evaluated. It may be more important to reduce the side effects of SC therapy than to improve its efficacy.

## 6. Clinical Application of SC Therapy for SCI

Many different types of SCs for the treatment of SCI have been used in various clinical trials with the primary goal of treating neurologically relevant diseases and injuries up to date. At present, clinical trials of SC transplantation for SCI are mostly concentrated in stage I-II.

Ra et al. studied the toxicity and tumorigenicity of human AD-MSCs. Eight male patients with SCI over 12 months received an intravenous injection of autologous AD-MSCs (4 × 10^8^ cells). During the 3-month follow-up period, all patients had no serious adverse events associated with transplantation [165]. Curtis et al. conducted a phase I clinical trial of human spinal cord-derived NSC transplantation for chronic SCI. In this trial, four patients with T2-T12 SCI underwent treatment including laminectomy, laminectomy, and dural incision, followed by a midline bilateral stereotactic injection of SCs for 6 times. All subjects did not experience serious adverse events 18-27 months after transplantation. The International Standards for Neurological Classification of Spinal Cord Injury (ISCNCCI) scores showed improved neurological function in one or two spinal segments [166]. A phase I nonrandomized controlled clinical trial conducted by Mendonca et al. enrolled 14 patients with chronic traumatic SCI (more than 6 months) who underwent autologous BM-MSCs injected into the lesion after laminectomy and dural incision. Baseline somatosensory evoked potentials (SSEP), magnetic resonance imaging (MRI), and urodynamics were evaluated before and after treatment. Pain scores were performed using the McGill Pain Questionnaire (MPQ) and the Visual Analog Scale (VAS). The results showed that autologous BM-MSC transplantation is safe and feasible in patients with chronic complete SCI and may contribute to the improvement of neurological function [167]. Satti et al. studied the safety of intrathecal autologous bone marrow stromal cells in 9 patients with SCI, including 6 with chronic SCI and 3 with subacute SCI. Each patient received two or three injections with a median number of 1.2 × 10^6^ cells/kg body weight. No treatment-related adverse events were observed during follow-up [168]. Shin et al. evaluated the safety and efficacy of human NSC transplantation in the treatment of traumatic cervical SCI in a phase I/IIA nonrandomized controlled clinical trial. In the 19 patients who underwent transplantation, there was no evidence of syringomyelia or tumor formation, deterioration of neurological function, and neuropathic pain or spasm. Eight of the patients had an improvement in the American Spinal Injury Association Impairment Scale (AIS), compared with only one in the control group [169]. Ghobrial et al. demonstrated through 12 months of clinical follow-up that intramedullary injection of human NSCs for the treatment of chronic cervical SCI has better safety. At the end of follow-up, the five patients had different degrees of improvement in the Graded Redefined Assessment of Strength, Sensibility, and Prehension (GRASSP) scores and the ISNCSCI scores [170]. Other similar studies have yielded good results [60, 95, 171]. However, phase III clinical trials conducted by Oh et al. showed that only 16 of the SCI patients who underwent autologous BM-MSC transplantation had improved neurological function, although all patients had no adverse reactions associated with SC injection [172].

In addition, there are a number of clinical trials that have not yet been initiated, either in progress, or terminated or revoked for different reasons (underfunded, inadequate patient inclusion, business decisions, etc.). It should be pointed out that different clinical trials have large differences in the number of patients, SCI site, type, severity and stage, follow-up time, SC type, mode of administration, and dosage. It is difficult to compare with each other to draw a positive conclusion. There is still a lack of long-term, large-scale, multicenter, standardized randomized controlled clinical trial results. Therefore, further clinical trials are needed to explore the clinical efficacy of SC therapy.

**Figure 3:**
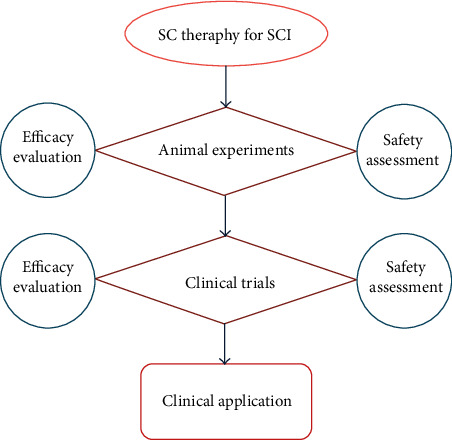
The application process of stem cells (SCs) for spinal cord injury (SCI). SC therapy for SCI must go through animal experiments and clinical trials before it can be applied in the clinic. During this period, the efficacy evaluation and safety assessment should be carried out.

The application process of SCs for SCI is shown in Figure 3. Clinical trials of SCs in SCI treatment are shown in Supplemental Table 1.

## 7. Pharmacological Activation of Endogenous SCs (ESCs)

Despite its great potential, the SC transplantation has not been widely used in the treatment of SCI for the current, due to the lack of donors, rejection reaction, and ethical factors. Notably, ESCs have been proved to be an effective substitution without all the mentioned problems. Exploring the methods of activating ESCs has become one of the hotspots concerned by scholars all over the world. VEGF plays an important role on nerve development. Cabezas et al. reported that the application of VEGF could promote the migration and differentiation of endogenous NSCs, thus achieving neuroprotection against SCI [173]. Fibroblast growth factor (FGF) is a critical factor in modulating angiogenesis and embryonic development. Kang et al. reported that intraperitoneal injection of FGF into SCI mice could effectively activate ESCs and increase the number of neurons. More importantly, the administration of FGF could promote ESCs and motor nerves, thus improving the motor function of mice [174]. Moreover, Khan et al. reported that SCI mice receiving the treatment of brain-derived neurotrophic factor (BDNF) demonstrated a better performance on motor function when compared with the control group [175]. In summary, the activation of ESCs could be an important and promising strategy for the treatment of SCI. Further research is needed in order to introduce novel and safe pharmacological interventions.

## 8. SC and Novel Approaches: Biomaterials, 3D Print, and Optogenetics

Recent studies indicated that SCs loaded with biological scaffolds could increase the survival rate of SCs, promote differentiation into neurons, increase the growth factor release, and improve axonal and myelin sheath regeneration [176]. Numerous studies indicated that SCI mice receiving biological scaffolds combined with SC transplantation demonstrated a better outcome on motor function than those that received SCs alone [177–179]. A growing number of studies indicated that 3D print might be another promising and exciting technology with huge potential for SCI treatment. The 3D print spinal implant contains dozens of tiny channels that are 200 *μ*m wide, which guide NSCs and axons to grow around the damaged spinal cord. Generally, a 3D printed spinal cord implant is composed of hydrogel and can be customized according to the individualized condition and can be quickly printed into implants of different sizes and shapes to accurately repair the spinal cord [180]. Koffler et al. filled the spinal cord implants with NSCs and then implanted them into the injured spinal cord like a jigsaw puzzle. New nerve cells begin to grow and synapses that transmit signals between nerve cells through axons begin to regenerate, ultimately connecting newborn nerve cells to each other and improving the repair of the spinal cord [181]. Optogenetics is the technology based on the introduction of photoactivatable proteins into physiologically or genetically defined cell populations using viral vectors [182]. Growing evidence suggests the potential effects of optogenetics combined with SCs on the treatment of SCI [182]. SCs cannot develop connections with other neurons, which are disrupted and keep in nonfunctional status due to the lack of regeneration at the injury site after SCI. Ahmad et al. reported that ChR2, a critical factor expressed on both motor neurons or SCs, allows the stimulation of neuronal activation and regeneration by illumination with blue light [183]. And many potential optogenetic tools targeting SC-associated signal, such as MAPK signal and PI3K/Akt/mTOR signal, were recently developed for the treatment of SCI [184, 185]. All those approaches mentioned above suggested that the development of new technologies to increase the efficacy of SCs may be a promising strategy for the treatment of SCI.

## 9. Conclusions and Prospects

In summary, SC therapy is a promising therapeutic strategy for SCI. However, there are still many problems that must be considered before the application of SC therapy, such as the issues that should be focused on include ethical issues, treatment effects, adverse reactions, complications, immune rejection, cell purification, and tumorigenicity. These disadvantages have brought huge treatment security risk. The detailed treatment strategies and mechanisms are still unclear, and how to control the side effects of the SC transplantation process is also a challenge and requires further exploration. In addition, due to the lack of large-scale clinical studies, most studies are conducted in animal models, and before being applied to clinical practice, SC therapy for SCI requires more animal experiments to be evaluated and then requires large-scale and multicenter clinical trial. Notably, targeting ESCs and combining SCs with new approaches might be the promising direction for the application of SC on SCI treatment in the future. It is believed that with the continuous development of SC technology, SC therapy will surely make a major breakthrough in the clinical treatment of SCI.

## Abbreviations

SCI: Spinal cord injury
SC: Stem cell
ROS: Reactive oxygen species
ESCs: Embryonic stem cells
ASCs: Adult stem cells
MSCs: Mesenchymal stem cells
HSCs: Hematopoietic stem cells
NSCs: Neural stem cells
iPSCs: Induced pluripotent stem cells
TSCs: Totipotent stem cells
PSCs: Pluripotent stem cells
USCs: Unipotent stem cells
DPSCs: Dental pulp stem cells
OECs: Olfactory ensheathing cells
ICH: Intracerebral hemorrhage
TBI: Traumatic brain injury
SAH: Subarachnoid hemorrhage
BM-MSCs: Bone marrow mesenchymal stem cells
HUC-MSCs: Human umbilical cord mesenchymal stem cells
AD-MSCs: Adipose-derived mesenchymal stem cells
BBB: Blood-brain barrier
IL: Interleukin
TNF-*α*: Tumor necrosis factor-*α*
CNS: Central nervous system
NT-3: Neurotrophins-3
GMCSF: Granulocyte-macrophage colony-stimulating factor
MAP2: Microtubule-associated protein 2
ARTN: Artemin
BDNF: Brain-derived neurotrophic factor
BBB: Basso, Beattie, and Bresnahan
GFAP: Glial fibrillary acidic protein
Bcl-2: B-cell lymphoma-2
GDNF: Glial cell-derived neurotrophic factor
VEGF: Vascular endothelial growth factor
bFGF: Basic fibroblast growth factor
TUNEL: Terminal deoxynucleotidyl transferase-mediated dUTP nick-end labeling
Bcl-xL: B-cell lymphoma-extra large
ISCNCCI: International standards for neurological classification of spinal cord injury
SSEP: Somatosensory evoked potentials
MRI: Magnetic resonance imaging
MPQ: McGill Pain Questionnaire
VAS: Visual analog scale
AIS: American spinal injury association impairment scale
GRASSP: Graded redefined assessment of strength, sensibility, and prehension
ESCs: Endogenous SCs.

## Supplementary Materials

Supplementary Materials Supplemental Table 1: clinical trials of SCs in the treatment for SCI.
