# Supplementary material for: Progress in Stem Cell Therapy for Spinal Cord Injury
Source: Stem Cells Int. 2020 Nov 5;2020:2853650. doi: 10.1155/2020/2853650 (PMC7661146; doi:10.1155/2020/2853650)
Supplement: Supplementary Materials — Supplemental Table 1: clinical trials of SCs in the treatment for SCI. [file 2853650.f1.docx]

Supplementary information

SupplementalTable 1. Clinical trials of SCs in the treatment for SCI
